# Methimazole Desensitization in a Patient Experiencing a Thionamide-induced Hypersensitivity Reaction

**DOI:** 10.1210/jcemcr/luae066

**Published:** 2024-05-27

**Authors:** Joseph Arguinchona, Avanika Mahajan, Alexei Gonzalez-Estrada, Eleanna De Filippis

**Affiliations:** Department of Medicine, Mayo Clinic Arizona, Scottsdale, AZ 85259, USA; Department of Medicine, Mayo Clinic Arizona, Scottsdale, AZ 85259, USA; Division of Allergy, Asthma and Clinical Immunology, Mayo Clinic Arizona, Scottsdale, AZ 85259, USA; Department of Endocrinology, Mayo Clinic Arizona, Scottsdale, AZ 85259, USA

**Keywords:** Graves disease, hyperthyroidism, methimazole, desensitization, hypersensitivity

## Abstract

Patients with newly diagnosed Graves disease often elect for treatment with the drug methimazole (MMI) over alternative therapies. However, MMI can commonly result in skin allergy that in severe cases can lead to discontinuation of therapy. We present a case of Graves thyrotoxicosis with a delayed hypersensitivity reaction while on MMI. The patient was successfully treated with a novel, individualized, 27-day desensitization protocol that resulted in tolerance of MMI with subsequent improvement in thyroid indices. Previous literature has offered various rapid desensitization protocols to MMI for immediate type hypersensitivity reactions. However, in nonimmediate, delayed hypersensitivity reactions, a slower desensitization protocol can be considered. As demonstrated in this case, desensitization to MMI is a reasonable alternative in patients who wish to avoid definitive therapy who develop an initial adverse reaction to MMI, as this can occur in up to 13% of treated cases.

## Introduction

Graves disease is an autoimmune process in which thyrotropin receptor antibody (TRAb) activates the TSH receptor resulting in a hyperthyroid state. Multiple modalities exist for management of Graves including antithyroid drugs (ATD), radioactive iodine ablation, and thyroidectomy. Patients with newly diagnosed Graves disease often choose treatment with the antithyroid medication methimazole (MMI) and wish to avoid definitive therapies (1, 2). Methimazole can be prescribed for prolonged periods (>5 years) with routine evaluation of thyroid indices to assess for return to and maintenance of a euthyroid state (3). Dosing should be titrated to the lowest effective dose to avoid unwanted side effects of the medication (2).

However, in up to 13% of treated cases, MMI can cause adverse reactions including skin rash, pruritus, urticaria, fever, nausea, and emesis that can result in discontinuation of the drug. In mild reactions, the ATD can be continued with a trial of concurrent antihistamine therapy. Additionally, propylthiouracil can be considered as an alternative agent, but up to 50% of patients experience cross sensitivity (4).

In such cases, desensitization protocols have been used with varying degrees of success (4, 5). Drug desensitization aims to achieve tolerance to the medication in patients who develop an initial reaction and can be utilized in both IgE- and non-IgE-mediated hypersensitivity reactions. Immediate, IgE-mediated hypersensitivity reactions typically use rapid desensitization protocols, whereas more delayed, cell-mediated hypersensitivity reactions use slow desensitization protocols (SDP) over days to weeks. Immediate drug hypersensitivity reactions occur at up to 6 hours (usually minutes to hours) after initial exposure, whereas delayed hypersensitivity reactions occur typically more than 6 hours after initial drug exposure (usually days to weeks) (6-8). On review of the literature, few SDP used for MMI intolerance have been described in detail (4).

Here, we present a patient who developed a delayed, cell-mediated reaction to MMI during treatment for Graves thyrotoxicosis. A novel, individualized, 27-day desensitization protocol was developed and resulted in tolerance to MMI with subsequent improvement in thyroid indices.

## Case Presentation

A 42-year-old female with a medical history of minimal change disease was referred to the endocrinology clinic for abnormal thyroid studies with associated insomnia, heat intolerance, palpitations, resting tremor, and weight loss.

## Diagnostic Assessment

Laboratory evaluation before her initial consultation revealed a TSH < 0.005 μIU/mL (reference, 0.450-4.500 μIU/mL), free thyroxine of 3.28 ng/dL (42.22 pmol/L; reference, 0.82-1.77 ng/dL [10.55-22.78 pmol/L]), triiodothyronine of 310 ng/dL (4.77 nmol/L; reference, 71-180 ng/dL [1.09-2.77 nmol/L]), fasting glucose 69 mg/dL (3.83 mmol/L; reference, 70-100 mg/dL [3.89–5.55 mmol/L]), as well as elevated thyroid peroxidase antibody, thyroid-stimulating immunoglobulin, and TRAb (Table 1). On examination, she had no evidence of thyroid eye disease.

**Table 1.** Thyroid laboratory studies at time of initial presentation and 3-months after completing the desensitization protocol seen in Table 2

Laboratory studies	TSH (0.450-4.500 μIU/mL)	fT4 (0.82-1.77 ng/dL [10.55-22.78 pmol/L])	TT3 (71-180 ng/dL [1.09-2.77 nmol/L])	TSI (0.00-0.55 IU/L)	TRAb (0.00-1.75 IU/L)	TPOAb (0-34 IU/mL)
During initial evaluation	<0.005 μIU/mL	3.28 ng/dL [42.22 pmol/L]	310 ng/dL [4.77 nmol/L]	0.67 IU/L	6.00 IU/L	62 IU/mL
At 3-mo follow-up after drug desensitization	6.080 μIU/mL	0.58 ng/dL [7.46 pmol/L]	87 ng/dL [1.33 nmol/L]	0.16 IU/L	1.39 IU/L	Not collected

Abbreviations: fT4, free thyroxine; TPOAb, thyroid peroxidase antibody; TRAb, thyroid receptor antibody; TSI, thyroid-stimulating immunoglobulin; TT3, total triiodothyronine

**Figure 1.**
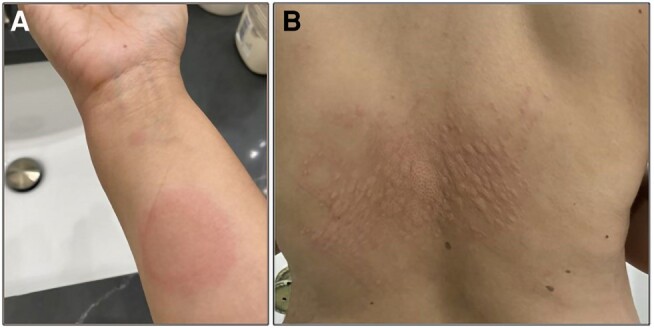
Macular exanthema shown here on the patient's right arm (A) and mid-back (B) that developed weeks after initiation of MMI.

A diagnosis of Graves disease was established. The risks and benefits of ATD therapy as well as definitive therapies were discussed with the patient. Although hesitant to start ATD, she agreed to begin MMI at a low dose of 10 mg daily. Laboratory tests were repeated 20 days later and both free thyroxine and triiodothyronine remained elevated. The patient agreed to double the dose of her methimazole to 10 mg twice a day, based on patient preference for twice as opposed to once daily dosing. Two weeks after increasing the dose, she developed a diffuse, pruritic maculopapular exanthema over her trunk, neck, and extremities (Fig. 1). MMI was stopped and her rash subsided in response to prednisone. Because of the delayed timing of the reaction, it was uncertain if the initial skin reaction was from MMI, or a coincidence. Given this, the medication was reinitiated with concurrent antihistamine therapy but unfortunately symptoms recurred on repeat exposure and the drug was stopped again.

## Treatment

The patient was invited to participate in the medical decision-making process and declined propylthiouracil because of concerns of hepatotoxicity and elected to avoid radioactive iodine therapy or surgery. Given this, with the assistance of an allergist, a novel, 10-step, 27-day desensitization protocol was developed specifically for her reaction to MMI with plans to taper prednisone once desensitization was completed (Table 2). This protocol involved dose increase every 3 days for a target dosage of 20 mg total daily on day 28. She was slowly titrated off systemic prednisone around 2 to 3 weeks after completing the MMI desensitization protocol. Overall, the patient tolerated prednisone therapy well apart from mildly increased anxiety levels.

**Table 2.** Proposed 10-step desensitization protocol with escalation of dose every third day for approximately 1 month

Steps	Daily Dose (mg)	Concentration/tablet	Amount	Days
1	0.01	0.01 mg/mL	1 mL	1-3
2	0.02	0.01 mg/mL	2 mL	4-6
3	0.04	0.01 mg/mL	4 mL	7-9
4	0.1	0.1 mg/mL	1 mL	10-12
5	0.2	0.1 mg/mL	2 mL	13-15
6	1	0.1 mg/mL	10 mL	16-18
7	2	0.1 mg/mL	20 mL	19-21
8	5	10-mg tablet	0.5 tablet daily	22-24
9	10	10-mg tablet	1 tablet daily	25-27
10	20	10-mg tablet	1 tablet twice daily	≥28

Initial dilutions were made by outpatient pharmacy by dissolving 10-mg tablets of methimazole in water.

## Outcome and Follow-up

Two days after completing the protocol, she developed a small pruritic papule on her left lower extremity that was mild compared with her prior reaction and resolved with high-dose antihistamines. Following this, she had no further adverse skin reactions on continued MMI therapy.

Three months after completing the protocol, she was reevaluated, noted to be tolerating MMI, 15 mg daily without further adverse reactions and tapered off prednisone. MMI dosing was reduced because of slight oversuppression of thyroid levels with a free thyroxine level of 0.58 ng/dL (7.47 pmol/L; reference, 0.82-1.77 ng/dL [10.55-22.78 pmol/L]) (Table 1). Subsequently, she had complete normalization of thyroid indices with an undetectable TRAb and thyroid-stimulating immunoglobulin. She continued to tolerate MMI without adverse reaction. Notably, the patient was started on low-dose thyroid replacement during this time in the setting of a continued medically induced hypothyroid state. Although again lowering of the dose of MMI would typically be the preferred approach in this setting, it was opted to instead keep the dose of MMI at the known effective dose and replace with levothyroxine. This was an anecdotal approach because in discussion with the allergist, there was concern that if MMI was further lowered, and then in the future had to be raised again because of elevation of antibody titers, this would again require a slow titration with concern for return of hypersensitivity reaction.

## Discussion

Modalities for the treatment of hyperthyroidism include radioactive iodine, surgery, and thionamide drug therapy. Antithyroid drug therapy has been used for 7 decades with the goal of achieving a euthyroid state. In practice, around half of patients treated with 12 to 18 months of ATD therapy will achieve remission, with remission defined as euthyroidism 3 to 6 months after the last administered dose of ATD. Most of these patients will remain euthyroid once normal thyroid indices are achieved (9, 10). Current guidelines suggest that treatment approach in hyperthyroidism should be individualized and part of a shared decision-making process with each patient. As van Kinschot et al. demonstrated, remission rate was the most important factor for both patient and provider in choice in initial therapy for Graves disease followed by type of therapy, with both physicians and patients initially preferring ATD therapy over alternative strategies (9).

A common deterrent to thionamide drug therapy including MMI in the treatment of Graves disease is drug-induced hypersensitivity reactions. Patients treated with ATD can develop adverse reactions ranging from minor skin reaction and gastrointestinal distress to, rarely, agranulocytosis in severe cases (4, 5, 9). In instances of benign skin hypersensitivity reactions, desensitization to the drug can be considered if it remains the therapy of choice by both the patient and physician. Drug desensitizations are contraindicated in severe adverse cutaneous drug reactions (SCARs). Severe cutaneous adverse reactions include Stevens-Johnson syndrome/toxic epidermal necrolysis, acute general exanthematous pustulosis, and drug reaction with eosinophilia and systemic symptoms. As a general approach all suspected causal agents should be avoided in SCARs and these cases should be referred to an allergist or dermatologist to manage these reactions (11).

**Figure 2.**
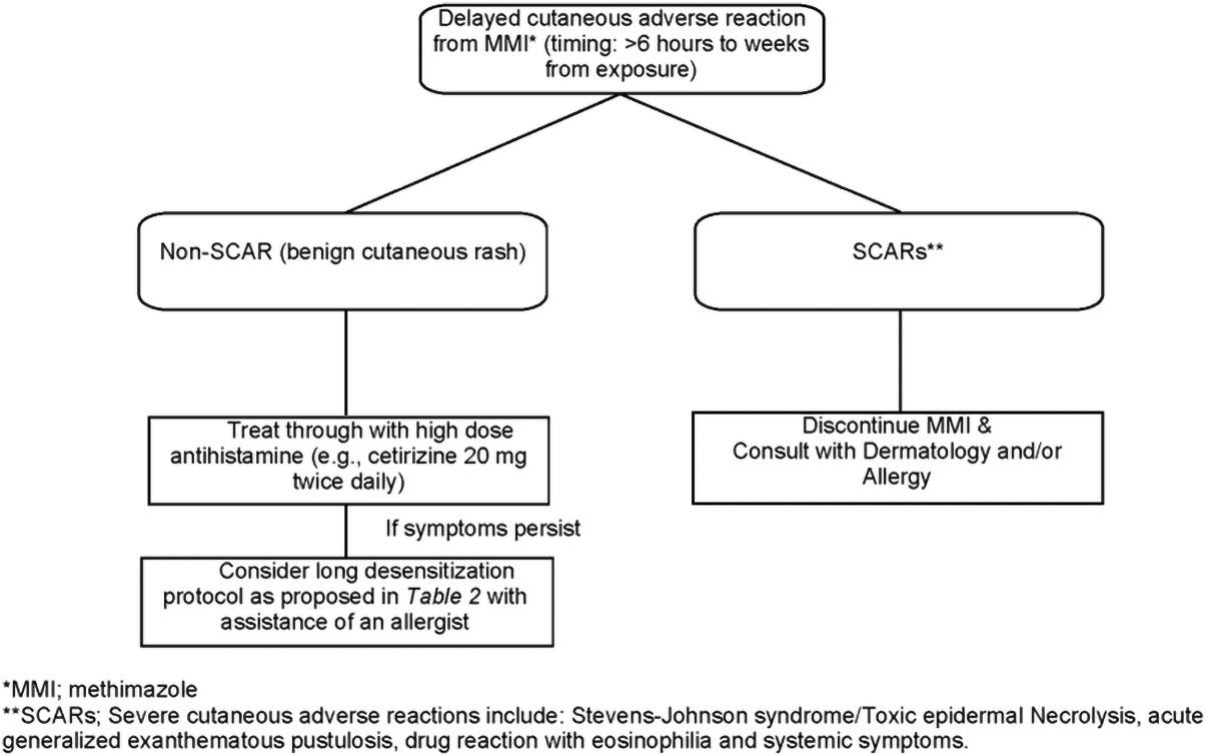
Proposed treatment algorithm for a delayed cutaneous adverse reaction to MMI.

As seen in this case, delayed reaction to drug therapy can begin anywhere from days to weeks after initiation to treatment and typically involves the activation and proliferation of T cells. Desensitization is a way of gradual uptitration of the drug dosage resulting in eventual drug tolerance. Mazhari et al. previously performed a retrospective review of 7 patients who developed side effects to MMI therapy. In this case series, the protocols proposed for desensitization were more consistent for immediate hypersensitivity reactions unlike the delayed reaction seen in our patient because they typically involved more rapid uptitration, often doubling the dose of the medication every 15 minutes until a therapeutic level of the drug was achieved (4, 6, 7). The proposed protocol in our case contrasts this involving uptitration of MMI over the course of nearly 1 month. To our knowledge, the literature remains scant on clearly outlined SDP for delayed type hypersensitivity reactions to MMI as used in our patient. Figure 2 summarizes a proposed treatment algorithm for patients treated with MMI who develop a cutaneous reaction.

This case demonstrates that with the assistance of an allergist, desensitization to MMI is a viable option in the setting of a known hypersensitivity reaction when it is the first-line treatment and no equally efficacious alternative treatments exist. In addition, patients’ preferences should also be considered during medical decision-making. In delayed, benign, hypersensitivity reactions, excluding SCARs, a slower desensitization protocol can be considered, as demonstrated by the 27-day protocol used in our case (Table 2).

## Learning Points

- Desensitization to MMI is a consideration in the setting of a known hypersensitivity reaction to the drug.
- When the reaction to MMI is a delayed (developing over days to weeks) benign rash, as in this case, a slow drug desensitization protocol can be considered.
- Patients’ preferences should be considered during the medical decision-making process pertaining to modality of treatment in Graves disease.

## Data Availability

Data sharing is not applicable to this article as no datasets were generated or analyzed during the current study.

## Abbreviations

ATD: antithyroid drug
MMI: methimazole
SCAR: severe adverse cutaneous drug reaction
SDP: slow desensitization protocol
TRAb: thyrotropin receptor antibody
